# Peripheral blood endothelial microparticles (CD144+) as a surrogate marker for coronary microcirculatory dysfunction in patients with stable coronary artery disease

**DOI:** 10.3389/fcvm.2026.1900067

**Published:** 2026-07-28

**Authors:** Dao-Kuo Yao, Xiao-Song Ding, Bing Hua, Xiao-Quan He, Lei Liu, Xian-Tao Song

**Affiliations:** Department of Cardiology, Beijing Friendship Hospital, Capital Medical, University, Beijing, China

**Keywords:** biomarker, coronary artery disease, coronary microvascular dysfunction, endothelial microparticles, index of microcirculatory resistance

## Abstract

**Objectives:**

In stable coronary artery disease (CAD), coronary microvascular dysfunction (CMD) is a prevalent cause of myocardial ischemia independent of epicardial stenoses. Although the index of microcirculatory resistance (IMR) is the invasive reference standard for CMD assessment, its routine use is limited by procedural demands and cost. We investigated whether circulating endothelial microparticles (EMPs, CD144+) correlate with IMR and could serve as a non-invasive surrogate for CMD in stable CAD.

**Methods:**

Fifty patients with stable CAD and 20 controls with normal angiograms underwent invasive physiological assessment, including IMR, fractional flow reserve (FFR), and coronary flow reserve (CFR), using a pressure-temperature sensor-tipped guidewire. Peripheral venous blood was analyzed for CD144+ EMPs by flow cytometry. Associations between EMPs and physiological indices were evaluated, with diagnostic utility assessed via ROC curve analyses.

**Results:**

Demographic and clinical profiles were similar across groups (all *P* > 0.05). Circulating EMP counts were markedly higher in CAD patients than controls (145.62 ± 101.35 vs. 119.98 ± 71.93 particles/*μ*L, *P* < 0.001), paralleled by elevated IMR values (22.14 ± 7.42 vs. 16.4 ± 4.41, *P* = 0.002). Within the CAD cohort, EMP levels correlated positively with IMR (*r* = 0.684, *P* < 0.001), but not in controls (*r* = 0.066, *P* = 0.783). Neither FFR nor CFR was associated with EMPs. Patients with IMR ≥ 23 had significantly higher EMP concentrations than those with IMR < 23 (*P* < 0.001). For discriminating CMD (IMR ≥ 23), EMPs yielded an AUC of 0.76 (95% CI: 0.62–0.89); at the optimal threshold of 130 particles/μL, sensitivity was 70.0%, specificity 70.6%, and negative predictive value 85.7%.

**Conclusions:**

Circulating CD144+ EMPs strongly correlate with invasive IMR in stable CAD. The moderate ROC performance (AUC 0.76) and high NPV suggest that peripheral EMP measurement holds promise as a non-invasive screening tool for CMD. However, these findings are exploratory and require prospective validation in larger, multi-center cohorts before clinical translation.

## Highlights

- Plasma CD144+ endothelial microparticles (EMPs) are significantly elevated in patients with stable coronary artery disease.
- Circulating EMPs are moderately to strongly correlated with invasive index of microcirculatory resistance (IMR) in stable CAD.
- ROC analysis shows moderate diagnostic performance (AUC: 0.76) for identifying CMD, with high negative predictive value (85.7%).
- Peripheral EMPs may serve as a potential non-invasive biomarker for coronary microvascular dysfunction pending validation in larger cohorts.

## Introduction

Worldwide and in China, CAD continues to rank first among causes of cardiovascular death and disability (1). Historically, epicardial stenosis was considered the primary driver of myocardial ischemia in CAD. Yet a growing body of evidence now implicates coronary microvascular dysfunction (CMD) as a key contributor to ischemic symptoms, adverse events, and prognosis in stable CAD patients, even when no severe obstructive lesion is present (2, 3). Indeed, CMD has gained recognition as an integral component of ischemic heart disease, conferring independent risk for major adverse cardiovascular outcomes (4).

As an invasive hemodynamic index, the index of microcirculatory resistance (IMR) has been validated for its specificity to coronary microvascular function (4). Unlike CFR, IMR is relatively insensitive to epicardial disease, thus affording a more direct measure of microvascular integrity (6). Prior data indicate that roughly 25% of stable CAD patients exhibit an elevated IMR, suggestive of occult microvascular injury (5, 7). Yet the requirement for catheterization and a pressure wire precludes its routine deployment, particularly in outpatient settings. Hence, a non-invasive surrogate marker that is readily measurable would carry considerable clinical appeal.

Several systemic biomarkers—including endothelial progenitor cells, ADMA, sVCAM-1, sICAM-1, and von Willebrand factor—have been examined for links to CMD and endothelial perturbation (8–10). Nonetheless, their performance has been inconsistent, and they tend to reflect generalized vascular inflammation rather than coronary-specific microvascular pathology. Endothelial microparticles (EMPs), membrane fragments shed from endothelial cells during activation, apoptosis, or injury (11), constitute a distinct class of biomarkers with potential advantages. They directly bear the surface antigens of their parent cells and provide a snapshot of endothelial injury at the moment of release. Recognized as sensitive indicators of vascular damage, EMPs have been found elevated in acute coronary syndromes, heart failure, hypertension, and diabetes (12, 13). Among endothelial markers, CD144 (VE-cadherin) is endothelial-specific, and CD144+ EMPs are routinely employed to identify endothelial-derived microparticles (14). However, whether EMPs add value beyond existing circulating markers specifically for CMD assessment—especially in stable CAD patients with invasive physiological data—remains less explored.

Although EMPs are closely tied to endothelial function, the direct link between peripheral EMP levels and IMR-derived microvascular status in stable CAD has not been clarified. Accordingly, the present work set out to examine the correlation between circulating CD144+ EMPs and IMR, and to assess the potential of EMPs as a non-invasive biomarker for CMD in this population.

## Methods

### Study population

The research protocol received approval from the Institutional Review Board of Beijing Friendship Hospital, Capital Medical University, and complied with the Declaration of Helsinki. Written informed consent was obtained from every participant before enrollment. This trial is registered at the Chinese Clinical Trial Registry (ChiCTR-ROC-17010412).

We prospectively enrolled 50 patients with stable CAD who were scheduled for coronary angiography and physiological assessment. In parallel, 20 controls—individuals presenting with chest pain or suspected CAD but found to have normal coronary arteries (no stenosis >20% in any major epicardial vessel) and a normal IMR—were recruited. The sample size was dictated by the number of eligible patients available over the recruitment window; all consenting and eligible patients were included consecutively. The control-to-case ratio of 1:2.5 was adopted on the basis of feasibility and mirrors ratios employed in analogous biomarker investigations.

Exclusion criteria encompassed: secondary ischemic heart disease; acute infection, connective tissue disorders, malignancy, or hematological diseases; severe hepatic or renal insufficiency; recent surgery, trauma, or cerebral hemorrhage; and glucocorticoid or immunosuppressant use within the preceding 3 months.

### Coronary physiological assessment

A pressure-temperature sensor guidewire (PressureWire Certus™, Abbott Park, Illinois, United States) was used to record coronary hemodynamic variables. After diagnostic angiography, the wire underwent *in-vivo* calibration and equalization to aortic pressure. To minimize vasomotor tone, 200 μg of intracoronary nitroglycerin was given. Hyperemia was provoked by intravenous adenosine triphosphate at 140 μg/kg/min. FFR was derived as the ratio of distal to aortic pressure during maximal hyperemia, with an abnormal threshold ≤0.80. CFR was calculated as the ratio of resting to hyperemic mean transit time (Tmn), abnormal ≤2.0. IMR was computed as Pd × hyperemic Tmn, with an abnormal value ≥23.

### Measurement of endothelial microparticles

Venous blood was drawn after an overnight fast and collected into 3.2% sodium citrate tubes. Platelet-free plasma was obtained through two sequential centrifugation steps: initially at 1,500×*g* for 15 min (4 °C) to pellet cells and platelets, then at 13,000×*g* for 2 min to generate platelet-depleted plasma, which was stored in aliquots at −80 °C until assayed.

Flow cytometric quantification of EMPs was performed on a BD FACSCalibur (BD Biosciences, San Jose, CA, USA) fitted with a 488 nm argon laser and a 635 nm red diode laser, with FSC and SSC in logarithmic mode. Daily instrument calibration relied on CaliBRITE beads (BD Biosciences). For staining, 100 μL of platelet-free plasma was mixed with 2 μL (1:50 dilution) of PE-conjugated mouse anti-human CD144 (VE-cadherin) antibody (clone 55-7H1, BD Biosciences) and incubated in the dark for 30 min at 4 °C. A PE-conjugated mouse IgG1 isotype (BD Biosciences) served as the negative control. After incubation, samples were diluted with 400 μL of PBS before acquisition.

Gating was performed as follows: P1 on the FSC/SSC plot removed debris and aggregates; P2 selected events with FSC < 1 μm, using 0.8 μm latex beads (Sigma-Aldrich) as internal size references; P3 defined CD144-PE positive events within the P2 gate. At least 50,000 events were collected per tube. EMPs were operationally defined as CD144+ particles <1 μm, and absolute counts (particles/μL) were derived using TruCount tubes (BD Biosciences) for bead-based quantification. All flow operators were blinded to clinical and angiographic data. The intra-assay coefficient of variation (10 replicates from a single sample) was 7.8%, and the inter-assay CV (5 measurements on separate days) was 12.3%.

**Figure 1:**
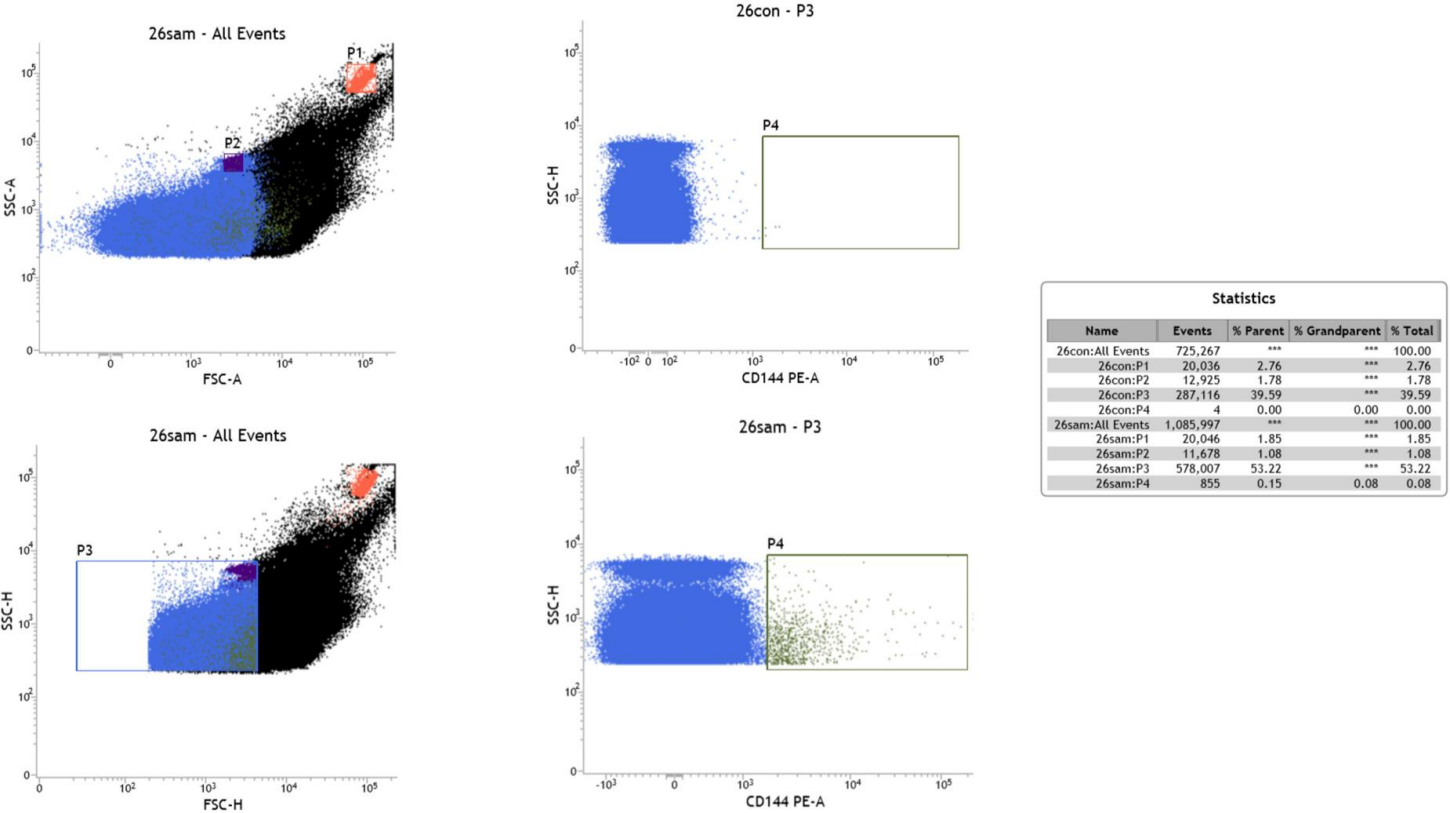
Gating strategy for identification of CD144-positive endothelial microparticles (EMPs) using flow cytometry. All events from the sample group (26sam) were first visualized with forward scatter and side scatter to define populations P1 and P2. Population P3 was further gated for subsequent phenotypic analysis. CD144 expression was evaluated in events within gate P3 from the control group (26con) and sample group (26sam), and CD144-positive EMPs were identified within gate P4. The statistics table summarizes event counts and corresponding percentages for each gated population in 26con and 26sam.

We chose CD144 (VE-cadherin) as the sole endothelial marker because it is constitutively expressed on endothelial cells, remains stable on microparticles irrespective of activation or apoptotic status, and has been well validated in numerous microparticle studies. Although additional markers (e.g., CD31, CD62E, CD146) might provide supplementary information, CD144+ EMPs capture the overall endothelial-derived microparticle pool and have shown the strongest and most reproducible associations with vascular injury in cardiovascular cohorts. Representative flow cytometry plots are provided in Figure 1.

### Statistical analysis

Continuous data are summarized as mean ± SD, and categorical data as counts (percentages). For group comparisons, Student's *t*-test was applied to continuous variables, while categorical variables were assessed via *χ*^2^ or Fisher's exact test, as appropriate. Spearman's rank correlation was used to evaluate bivariate associations. A *post-hoc* power calculation (G*Power 3.1.9.7) indicated that with the observed correlation of *r* = 0.684 between EMPs and IMR in the CAD group, *α* = 0.05 (two-tailed), and power = 0.80, the minimum required sample was 15 participants. Hence, our CAD cohort of 50 patients provided sufficient power for the primary correlation analysis. Nevertheless, this power analysis was performed retrospectively; the study was not prospectively powered for subgroup or multivariable modeling.

For multivariable analysis, we constructed a linear regression model with EMP level as the dependent variable—consistent with our objective of testing whether a readily obtainable peripheral biomarker could mirror invasive IMR. The independent predictors entered were IMR, age, sex, hypertension, diabetes, and statin use. Multicollinearity was checked using variance inflation factors (all VIFs < 2.0). Model assumptions were confirmed through partial regression plots (linearity), Shapiro–Wilk test (residual normality, *P* = 0.132) and *Q*–*Q* plots, Breusch–Pagan test (homoscedasticity, *P* = 0.218), and Cook's distance (no influential outliers, all values <1.0).

ROC curves were constructed to appraise the diagnostic accuracy of EMPs for distinguishing CAD from controls and for identifying CMD (IMR ≥ 23). A two-sided *P* < 0.05 defined statistical significance. All analyses were executed with SPSS 21.0 (IBM Corp.) and MedCalc version 20.0 (MedCalc Software).

## Results

### Baseline characteristics

No significant between-group differences emerged for age, sex, hypertension, diabetes, hypercholesterolemia, smoking history, or family history of CAD (all *P* > 0.05), indicating adequate clinical matching. As expected, aspirin, statin, and *β*-blocker use was more frequent in the CAD arm (*P* < 0.001 for each). Full details are summarised in Table 1.

**Table 1:** Baseline characteristics of stable CAD patients and control subjects.

Characteristic	Stable CAD (*n* = 50)	Control (*n* = 20)	*P* value
Age, years	62.4 ± 7.4	58.3 ± 8.2	0.652
Male, *n* (%)	32 (64)	13 (65)	0.937
Hypertension, *n* (%)	28 (56)	11 (55)	0.939
Diabetes mellitus, *n* (%)	20 (40)	7 (35)	0.698
Hypercholesterolemia, *n* (%)	32 (64)	14 (70)	0.633
Current smoker, *n* (%)	13 (26)	5 (25)	0.931
Family history of CAD, *n* (%)	6 (12)	2 (10)	0.812
Chronic renal dysfunction, *n* (%)	2 (4)	0 (0)	0.364
Stroke, *n* (%)	1 (2)	0 (0)	0.524
Peripheral arterial disease, *n* (%)	7 (14)	1 (5)	0.285
Medications			
Aspirin	50 (100)	12 (60)	<0.001
Statins	50 (100)	14 (70)	<0.001
ACEI/ARBs	28 (56)	11 (55)	0.939
*β*-receptor blockers	42 (84)	6 (30)	<0.001
CCBs	10 (20)	4 (20)	1.000

Data are presented as mean ± standard deviation (SD) or *n* (%), unless otherwise indicated. CAD, coronary artery disease; ACEI/ARB, angiotensin-converting enzyme inhibitor/angiotensin receptor blocker; CCB, calcium channel blocker.

### Comparison of coronary physiological parameters

FFR proved significantly lower among stable CAD patients (0.82 ± 0.05 vs. 0.96 ± 0.02, *P* < 0.001), whereas IMR was higher in this group (22.14 ± 7.42 vs. 16.4 ± 4.41, *P* = 0.002). The proportion of individuals with an IMR ≥ 23 was larger in the CAD cohort (32.0% vs. 10.0%). CFR did not differ appreciably between groups (*P* = 0.079) (Table 2).

**Table 2:** Coronary physiological parameters in stable CAD and control groups.

Parameter	Stable CAD (*n* = 50)	Control (*n* = 20)	*P* value
FFR	0.82 ± 0.05	0.96 ± 0.02	<0.001
FFR > 0.80, *n* (%)	32 (64.0)	20 (100)	<0.001
FFR ≤ 0.80, *n* (%)	18 (36.0)	0 (0)	—
CFR	3.05 ± 0.98	3.51 ± 0.96	0.079
CFR ≥ 2.0, *n* (%)	39 (78.0)	18 (90.0)	0.243
CFR < 2.0, *n* (%)	11 (22.0)	2 (10.0)	—
IMR	22.14 ± 7.42	16.40 ± 4.41	0.002
IMR ≥ 23, *n* (%)	16 (32.0)	2 (10.0)	0.057
IMR < 23, *n* (%)	34 (68.0)	18 (90.0)	—

Data are presented as mean ± SD or *n* (%). FFR, fractional flow reserve; CFR, coronary flow reserve; IMR, index of microcirculatory resistance. Abnormal cutoffs: FFR ≤ 0.80, CFR ≤ 2.0, and IMR ≥ 23.

### Circulating EMP levels across groups

Plasma EMP counts were notably elevated in stable CAD relative to controls (145.62 ± 101.35 vs. 119.98 ± 71.93 particles/μL, *P* < 0.001). Within the CAD group, patients with IMR ≥ 23 displayed higher EMP concentrations than those with IMR < 23 (156.31 ± 71.61 vs. 138.93 ± 57.85, *P* < 0.001). No significant EMP differences were observed across FFR- or CFR-defined subgroups (Table 3).

**Table 3:** Circulating EMP levels in subgroups.

Subgroup	Stable CAD (particles/μL)	*P* value	Control (particles/μL)	*P* value
Average	145.62 ± 101.35	<0.001^†^	119.98 ± 71.93	—
FFR > 0.8	139.61 ± 78.34	0.64	119.98 ± 71.93	—
FFR ≤ 0.8	147.45 ± 63.57	—	NA	—
CFR ≥ 2.0	142.32 ± 52.38	0.23	116.78 ± 67.34	0.82
CFR < 2.0	149.79 ± 76.53	—	121.39 ± 79.11	—
IMR ≥ 23	156.31 ± 71.61	<0.001	123.38 ± 71.35	0.67
IMR < 23	138.93 ± 57.85	—	117.62 ± 59.05	—

Data are mean ± SD. EMPs, endothelial microparticles; CAD, coronary artery disease; FFR, fractional flow reserve; CFR, coronary flow reserve; IMR, index of microcirculatory resistance. *P* values denote comparisons between the indicated subgroups within the same column (e.g., FFR > 0.8 vs. FFR ≤ 0.8; IMR ≥ 23 vs. IMR < 23).

† *P* value for comparison between the overall stable CAD average and control average.

### Correlations between EMPs and invasive physiological indices

**Figure 2:**
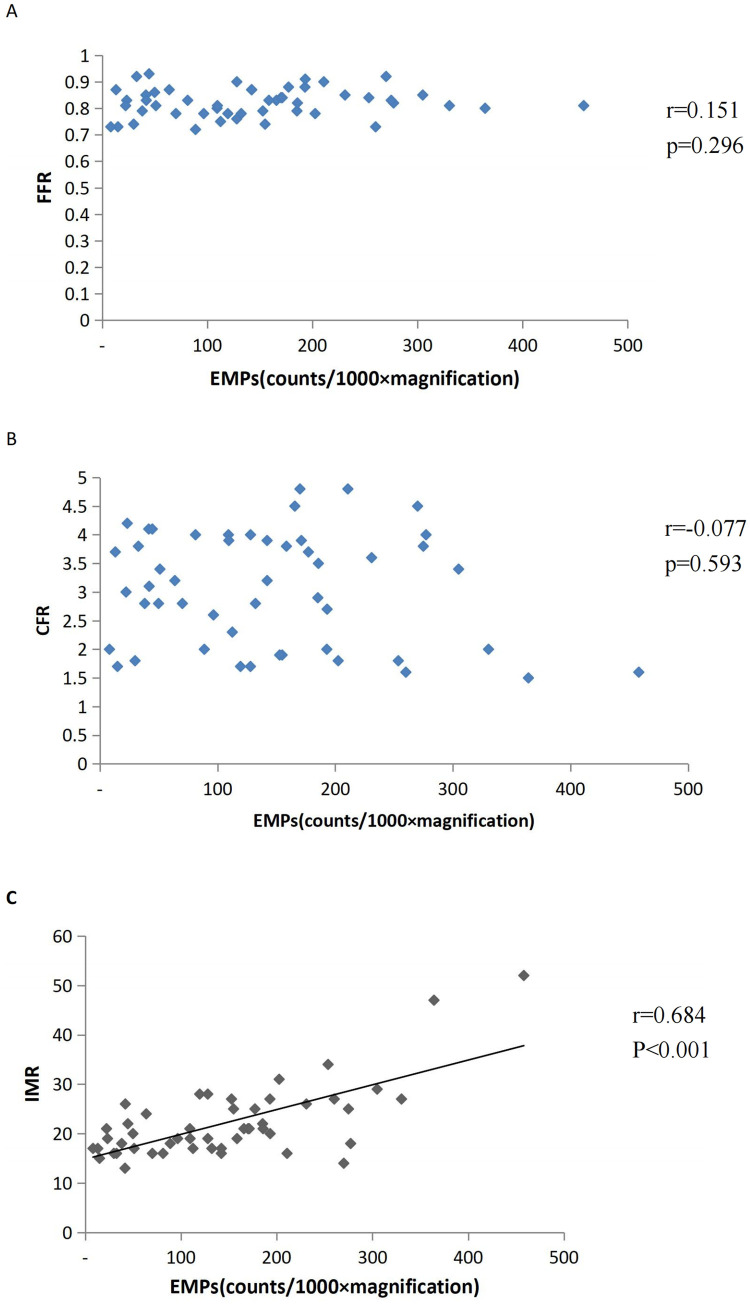
Correlations between circulating EMPs and coronary physiological indexes in stable CAD patients. **(A)** EMPs vs. FFR: *r* = 0.095, 95% CI: −0.184 to 0.363, *P* = 0.513. **(B)** EMPs vs. CFR: *r* = −0.143, 95% CI: −0.398 to 0.132, *P* = 0.322. **(C)** EMPs vs. IMR: *r* = 0.684, 95% CI: 0.504–0.806, *P* < 0.001.

In the stable CAD population, EMP levels correlated positively with IMR (Spearman *r* = 0.684, 95% CI: 0.504–0.806, *P* < 0.001), reflecting a moderate-to-strong association. The coefficient of determination (*R*^2^ = 0.468) indicates that EMPs explain roughly 46.8% of IMR variance, leaving substantial residual variability. By contrast, no such correlation existed in the control group (*r* = 0.066, *P* = 0.783). Neither FFR nor CFR showed meaningful correlations with EMPs in either cohort (Table 4, Figure 2).

**Table 4:** Multivariable linear regression analysis of factors associated with circulating EMP levels in stable CAD patients (*n* = 50).

Variable	Unstandardized *β*	95% CI	Standardized *β*	*P* value
IMR (per 1 unit increase)	6.12	3.45–8.79	0.498	<0.001
Age (per 1 year)	0.78	−1.25–2.81	0.058	0.445
Male sex (vs. female)	−4.90	−27.50–17.70	−0.032	0.672
Hypertension (vs. no)	11.20	−12.30–34.70	0.085	0.348
Diabetes mellitus (vs. no)	7.90	−15.80–31.60	0.061	0.512
Statin use (vs. no)	−22.60	−50.20–5.00	−0.168	0.108

The dependent variable was plasma EMPs (particles/μL). The model had an adjusted R² of 0.511 (*P* < 0.001). Variance inflation factor (VIF) values for all variables were <2.0. CI, confidence interval; EMPs, endothelial microparticles; IMR, index of microcirculatory resistance.

### Multivariable linear regression

With EMP level as the dependent variable and IMR, age, sex, hypertension, diabetes, and statin use entered as independent predictors, IMR maintained a significant independent association with EMPs after full adjustment (standardized *β* = 0.498, 95% CI: 0.285–0.711, *P* < 0.001). Among other covariates, statin use exhibited a trend toward lower EMPs that fell short of significance (*β* = −0.168, *P* = 0.108). The overall model accounted for 51.1% of EMP variability (adjusted *R*^2^ = 0.511, *P* < 0.001). Variance inflation factors remained below 2.0 for all variables, excluding meaningful collinearity. These findings suggest that the EMP–IMR relationship operates independently of traditional risk factors and commonly used medications (Table 4).

### Sensitivity analyses

To address potential confounding by differential medication use—particularly statins—we performed two additional analyses. Restricting the sample to participants on statin therapy (*n* = 64, including all 50 CAD patients and 14 controls), the EMP–IMR correlation persisted (*r* = 0.612, *P* < 0.001). In a second approach, we adjusted for propensity score-estimated likelihood of statin prescription (based on age, sex, LDL-cholesterol, and CAD diagnosis); after this adjustment, EMPs remained significantly tied to IMR (standardized *β* = 0.458, 95% CI: 0.224–0.692, *P* < 0.001). These sensitivity analyses reinforce the robustness of the primary association and argue against its being wholly attributable to medication imbalances.

As an internal check on stability, leave-one-out cross-validation within the CAD cohort yielded a mean correlation coefficient of *r* = 0.671 (range: 0.621–0.698), closely aligned with the original estimate (*r* = 0.684), further supporting the reliability of the observed correlation.

### Diagnostic performance of EMPs (ROC analysis)

**Figure 3:**
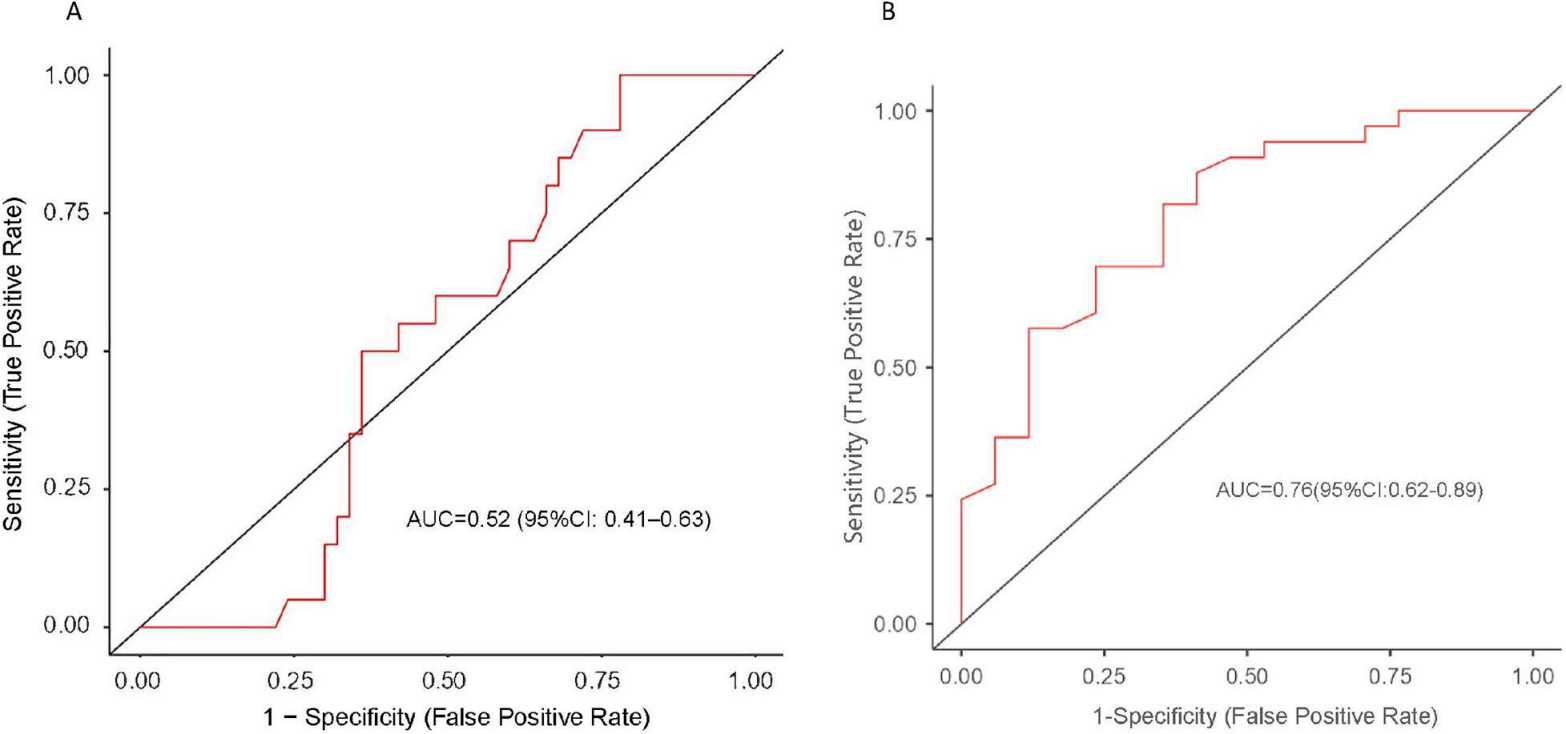
Receiver operating characteristic (ROC) curves for EMPs. **(A)** ROC curve for EMPs discriminating stable CAD from controls (AUC = 0.52, 95% CI: 0.41–0.63). **(B)** ROC curve for EMPs discriminating patients with IMR ≥ 23 (CMD) from those with IMR < 23 (AUC = 0.76, 95% CI: 0.62–0.89).

For discriminating stable CAD from controls, EMPs produced an AUC of 0.52 (95% CI: 0.41–0.63). At a cut-off of 128 particles/μL, sensitivity reached 55.0% and specificity 56.0%, with a positive predictive value of 75.7% and a negative predictive value of 33.2%. When applied to identify CMD (IMR ≥ 23), the AUC was 0.76 (95% CI: 0.62–0.89); at 130 particles/μL, sensitivity was 70.0%, specificity 70.6%, with a positive predictive value of 48.0% and a negative predictive value of 85.7% (Table 5, Figure 3). These results point to a potential role for EMPs as a rule-out test for CMD, given the high NPV, although the moderate AUC underscores that EMPs alone are insufficient for definitive diagnostic confirmation.

**Table 5:** Diagnostic performance of circulating EMPs for predicting CAD and coronary microvascular dysfunction (IMR ≥ 23).

Outcome	AUC	95% CI	Cut-off (particles/μL)	Sensitivity (%)	Specificity (%)	PPV (%)	NPV (%)
CAD vs. Control	0.52	0.41–0.63	128	55.0	56.0	75.7	33.3
CMD (IMR ≥ 23)	0.76	0.62–0.89	130	70.0	70.6	48.0	85.7

EMPs, endothelial microparticles; CAD, coronary artery disease; CMD, coronary microvascular dysfunction; IMR, index of microcirculatory resistance; AUC, area under the curve; CI, confidence interval; PPV, positive predictive value; NPV, negative predictive value.

## Discussion

Three principal observations emerge from this work. First, stable CAD patients exhibit both higher circulating CD144+ EMP levels and higher IMR than controls. Second, EMPs correlate moderately-to-strongly with IMR in the CAD cohort, an association that withstands adjustment for age, sex, cardiovascular risk factors, and statin use in both multivariable and sensitivity models. Third, the marked EMP elevation in patients with IMR ≥ 23, coupled with an ROC-derived NPV of 85.7%, suggests that EMPs may hold promise as a non-invasive screening marker for microvascular dysfunction, albeit with moderate overall diagnostic accuracy.

### Relation to previous work

Our observation of elevated EMPs in stable CAD echoes earlier reports (15). To our knowledge, however, this is the first investigation to directly correlate CD144+ EMPs with invasive IMR in stable CAD and the first to evaluate EMP diagnostic performance for CMD via ROC analysis. Prior studies have linked EMPs to endothelium-dependent vasodilation (flow-mediated dilation) or to hard cardiovascular events, but the specific connection with pressure-wire-derived microvascular resistance has not previously been addressed.

Interestingly, we found no association between EMPs and either FFR or CFR. This dissociation is conceptually consistent with the view that EMPs reflect systemic endothelial injury and microvascular integrity rather than focal epicardial stenosis (FFR) or global coronary flow reserve (CFR). Because IMR is widely considered a more specific metric of microvascular resistance, less confounded by epicardial disease (16), the positive EMP–IMR correlation lends support to the notion that endothelial damage and microvascular resistance are closely intertwined.

### Potential mechanisms

Given the cross-sectional design, causality cannot be inferred; still, several mechanistic hypotheses merit consideration. EMPs carry bioactive cargo—including reactive oxygen species, pro-inflammatory microRNAs, and adhesion molecules such as ICAM-1 and VCAM-1 (13)—that may impair nitric oxide bioavailability, promote vasoconstriction, and foster microvascular rarefaction (17, 18). EMP release occurs as a consequence of endothelial activation or apoptosis; under oxidative stress, elevated ROS can activate caspase-3 and Rho kinase pathways, prompting cytoskeletal remodelling and vesicle shedding (19, 20). Simultaneously, pro-inflammatory cytokines (e.g., TNF-*α*, IL-6), acting through NF-*κ*B, upregulate endothelial adhesion molecules, potentially amplifying both EMP generation and microvascular inflammation (21).

Recent insights into extracellular vesicle biology have moved beyond viewing EMPs as inert debris; they are now recognised as bioactive vectors capable of transferring miRNAs, proteins, and lipids to recipient cells, thereby modulating vascular tone and endothelial function (22, 23). It is plausible that EMP-mediated intercellular communication contributes to sustaining microvascular dysfunction in CAD (24). However, we emphasise that these interpretations remain speculative. Alternatively, elevated EMPs may simply parallel diffuse endothelial injury without directly participating in its pathogenesis (25). Our data demonstrate association, not causation.

### Clinical implications—with caveats

Should these findings be validated, measurement of peripheral EMPs might offer a non-invasive, low-cost screening avenue for CMD in stable CAD, particularly in outpatient settings where invasive IMR is unavailable. Yet the moderate AUC (0.73) cautions that EMPs alone are unlikely to serve as a standalone diagnostic; their main utility may lie in ruling out CMD, given the high NPV. A low EMP value could potentially reduce the need for unnecessary invasive procedures, but this remains hypothetical pending further study. EMPs might also emerge as a therapeutic target; statins, SGLT2 inhibitors, and exercise have been shown to lower EMPs in other populations, raising the question of whether EMP reduction translates into improved IMR and clinical outcomes (26, 27). A prospective multi-centre validation study is currently being planned to test these possibilities in a larger, independent cohort.

### Limitations

Several limitations warrant mention. This was a single-centre study with a modest total sample (*n* = 70, 50 CAD and 20 controls). Although *post-hoc* power analysis indicated adequate power for the primary correlation, the limited size restricts precision for subgroup estimates, precludes extensive subgroup exploration, and raises the possibility of overfitting in multivariate models. Correlation coefficients from small cohorts may be inflated, and independent replication is essential. Second, we measured only CD144+ EMPs; we did not distinguish activated (e.g., CD62E+) from apoptotic (e.g., annexin V+) subtypes, which may carry distinct biological implications. Third, acetylcholine provocation testing—which would have provided complementary information on endothelium-dependent microvascular function—was not performed. Fourth, the cross-sectional design precludes causal inferences. Fifth, the control group had lower statin and aspirin use; despite statistical adjustment and sensitivity analyses, residual confounding cannot be entirely excluded. Finally, the lack of an external validation cohort limits generalisability and precludes definitive recommendations on clinical use. These findings are hypothesis-generating and should be interpreted with appropriate caution.

## Conclusions

In stable CAD patients, circulating CD144+ endothelial microparticles correlate moderately-to-strongly with invasive IMR, independent of traditional risk factors and medications. ROC analysis indicates moderate diagnostic performance for CMD identification, with a high negative predictive value that supports a potential rule-out role. Peripheral EMPs may thus represent a candidate non-invasive biomarker for coronary microvascular dysfunction, useful for risk screening and possibly as a therapeutic target. However, larger, multi-centre prospective validation is mandatory before any clinical application can be endorsed.

## Data Availability

The original contributions presented in the study are included in the article/Supplementary Material, further inquiries can be directed to the corresponding author/s.
